# A case of midbrain germinoma: A literature review for radiographic and clinical features

**DOI:** 10.1093/noajnl/vdad043

**Published:** 2023-04-19

**Authors:** Yohei Miyake, Kensuke Tateishi, Akito Oshima, Takeshi Hongo, Kaishi Satomi, Koichi Ichimura, Ayumi Kato, Hiromichi Iwashita, Daisuke Utsunomiya, Tetsuya Yamamoto

**Affiliations:** Department of Neurosurgery, Graduate School of Medicine, Yokohama City University, Yokohama, Japan; Department of Neurosurgery, Graduate School of Medicine, Yokohama City University, Yokohama, Japan; Laboratory of Biopharmaceutical and Regenerative Science, Graduate School of Medical Science, Yokohama City University, Yokohama, Japan; Department of Neurosurgery, Graduate School of Medicine, Yokohama City University, Yokohama, Japan; Department of Neurosurgery, Graduate School of Medicine, Yokohama City University, Yokohama, Japan; Department of Pathology, Kyorin University School of Medicine, Tokyo, Japan; Deparment of Brain Disease Translational Research, Juntendo University Faculty of Medicine, Tokyo, Japan; Department of Radiology, Yokohama City University Hospital, Yokohama, Japan; Department of Pathology, Yokohama City University Hospital, Yokohama, Japan; Department of Radiology, Yokohama City University Hospital, Yokohama, Japan; Department of Neurosurgery, Graduate School of Medicine, Yokohama City University, Yokohama, Japan

**Keywords:** literature review, midbrain germinoma, molecular genetics

**Intracranial germinomas mainly occur in the neurohypophyseal trunk and pineal body but are less frequent in the basal ganglia. Other sites are rare and account for only 6% of primary lesions of germinomas.** **^1^ Among them midbrain germ cell tumors are extremely rare, with only 9 cases reported to date.** **^2–10^ Here, we present a case of germinoma of the midbrain that was diagnosed using histopathological and DNA methylation classifier analysis. Through a literature review, we summarized the clinical and radiographic characteristics of midbrain germinomas for diagnostic and therapeutic development.**

## Case Report

**Figure 1.**
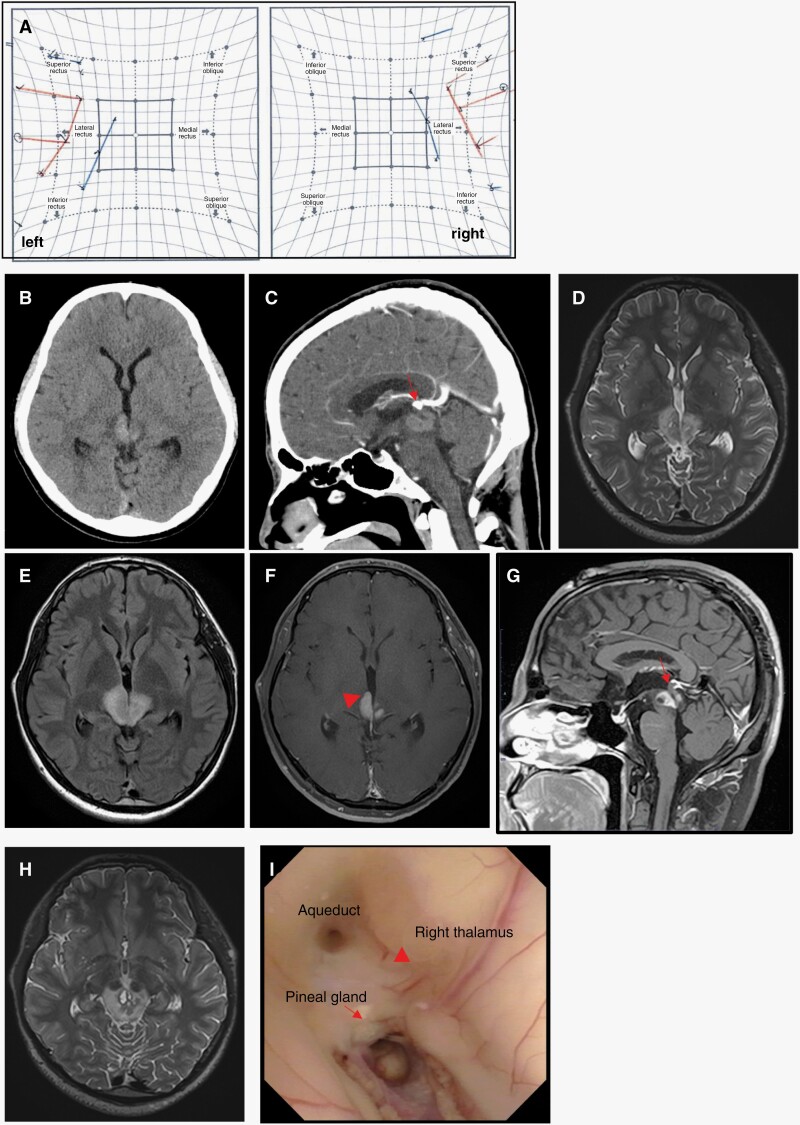
Preoperative and intraoperative findings. (A) Preoperative Hess screen test. (B, C) Computed tomography imaging (axial [B], sagittal [C]) showing high-density tumor at the dorsal midbrain. The pineal region with calcification is located at the upper edge of the tumor (arrow). (D, E) T2-weighted (D) and fluid-attenuated inversion recovery (E) magnetic resonance images showing iso- to slightly high-intensity lesion surrounded by hyperintense area. (F) Gadolinium-enhanced T1-weighted imaging demonstrating midbrain tumor extended to the medial of the bilateral thalamus (arrowhead). (G) Intact pineal gland (arrow). (H) T2-weighted imaging showing cystic lesions. (I) Operative findings. Right thalamic lesion (arrowhead) and calcified pineal region (arrow).

A 25-year-old man (YMG206), without relevant family/cancer history, complained of progressive bilateral ptosis and double vision for 6 months. Neurological examination revealed dilated pupils, loss of light reflexes, and ocular motility disorder without abduction, indicating bilateral oculomotor nerve palsy. Hess screen test showed that the ocular position was bilaterally abducens with severe adduction disorder (Figure 1A). No symptoms of diabetes insipidus were observed in this patient. Head computed tomography (CT) scans demonstrated a high-density area in the midbrain extending to the medial portion of the bilateral thalamus (Figure 1B and C). Hematological examinations did not reveal any abnormalities. Basal serum human chorionic gonadotropin beta-subunit, alpha-fetoprotein, and anterior and posterior pituitary-related hormonal values were normal. Magnetic resonance imaging (MRI) demonstrated an iso-intensity lesion on T1-weighted images, and iso- to slightly high-intensity lesion on T2-weighted and fluid-attenuated inversion recovery (FLAIR) images of the midbrain, mainly in the tegmentum. T2/FLAIR hyperintensity area extended to surrounding structures including the tectum and thalamus (Figure 1D and E). The lesion showed hyperintensity on diffusion-weighted images. Contrast-enhanced MRI demonstrated homogenous enhancement of the tumor corresponding to the T2/FLAIR high-intensity area (Figure 1F and G). A thin-slice contrast-enhanced MRI also revealed an intact pineal gland (Figure 1G). Cystic components were scattered throughout the tumor (Figure 1H). The pineal body with calcification was located at the upper edge of the tumor on sagittal view of contrast-enhanced CT image (Figure 1C). Collectively, we speculated that the tumor originated from the midbrain and preoperatively suspected a malignant brain stem glioma. The patient did not receive any treatment before surgery.

We performed an endoscopic biopsy and third ventriculostomy from the left frontal lobe. The medial side of the right thalamus, which corresponds to the biopsy site, showed a yellowish color change. (Figure 1I, see Supplementary Video). Macroscopically, the calcified pineal region was normal (Figure 1I). After diagnosis, described as below, the patient underwent chemotherapy with carboplatin and etoposide for 3 cycles along with radiation therapy. Radiation therapy consisted of whole-ventricle irradiation with a 0.5 cm margin (23.4 Gy in 13 fractions) with a focal boost to the tumor bed (12.6 Gy in 7 fractions, Supplementary Figure 1A). A complete response was achieved after the initial treatment (Supplementary Figure 1B). His symptoms improved, but oculomotor nerve palsy persisted partially 1 year after the initial diagnosis (Supplementary Figure 1C).

## Pathology and Molecular Diagnosis

**Figure 2.**
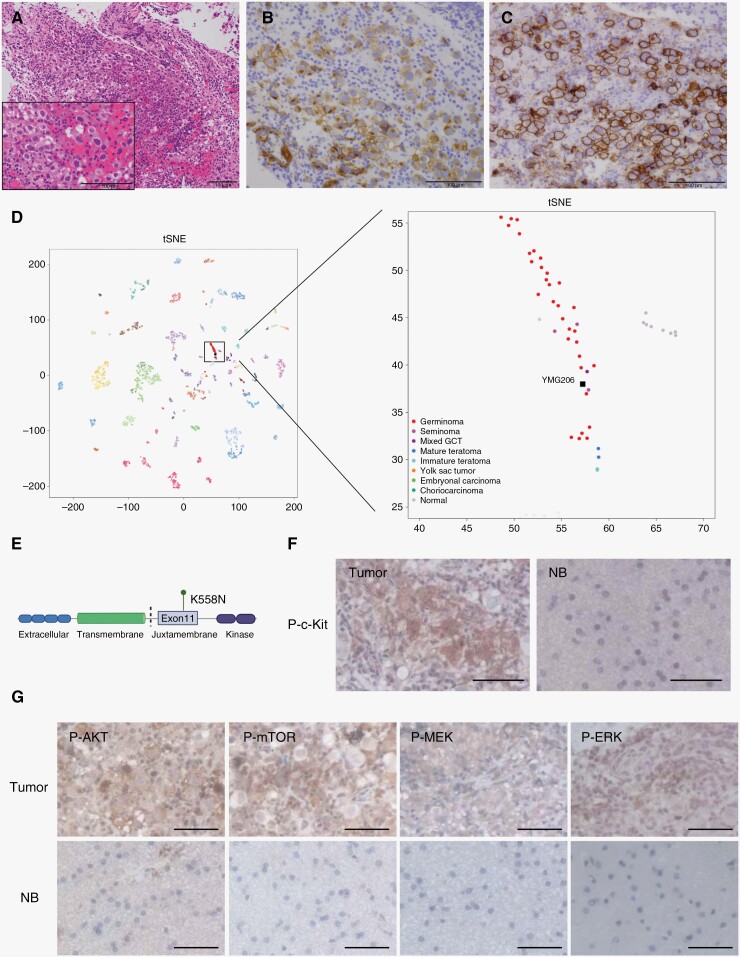
Histopathological, genomic, and epigenomic analysis. (A) Hematoxylin and eosin staining. Bars, 100 μm. (B, C) Immunostaining for c-kit (B) and D2-40 (C). Bars, 100 μm. (D) Unsupervised clustering using t-SNE analysis by using DNA methylation data. (E) A scheme of the KIT gene and single nucleotide variant in this tumor. (F, G) Immunohistochemistry for indicated proteins. NB, normal brain. Bars, 50 μm.

Pathological examination revealed 2 cell patterns composed of large tumor cells with clear cytoplasm, round nuclei, and prominent nucleoli, and a population of small lymphocytes, which were diffusely stained for CD45 and partially stained for CD3 and CD20 (Figure 2A; Supplementary Figure 2A). The tumor cells were positive for c-kit (Dako) and D2-40 (Nichirei; Figure 2B and C). Collectively, the tumor was diagnosed as a germinoma. We also performed genome-wide DNA methylation array (the Infinium MethylationEPIC v.1.0 BeadChip Kit, Illumina) and whole-exome sequencing (WES; Novogene) to examine genomic and epigenetic characteristics in this midbrain tumor. Unsupervised clustering using t-SNE analysis, as indicated by DNA methylation analysis, demonstrated that this tumor plotted close to the germinoma cluster (Figure 2D). Methylation classifier (ver. 12.5) revealed a classification matched to GCT_GERM_A (0.902688487; Supplementary Table S2). Together with the radiographic and intraoperative findings, we considered this germinoma originated in the midbrain. Chromosomal instability was not observed in this tumor (Supplementary Figure 2B).

WES identified a single nucleotide variant within *KIT* (c.1674G > C, *K558N*, juxtamembrane domain), which was annotated as an only pathogenic mutation by GATK MuTect2 (Figure 2E; Supplementary Table S1). To verify if *KIT K558N* is a pathogenic mutation of this tumor, we assessed protein expression status. As expected, phospho-c-kit was highly expressed in tumor cells as compared to normal control brain cells. In addition, phospho-AKT and -mTOR (phosphatidylinositol-3 kinase [PI3K] pathway), and phospho-MEK and -ERK (mitogen-activated protein kinase [MAPK] pathway), downstream pathways of c-kit protein, were also expressed within tumors (Figure 2F and G).

## Discussion

To date, 10 cases of midbrain germinomas, including the present case, have been reported. Among them, 9 cases were pure germinomas (Table 1),^2–9^ and 1 was a mixed germ cell tumor comprising mature teratoma and germinoma.^10^ Midbrain intrinsic tumors, such as pilocytic astrocytoma and diffuse midline glioma, H3 K27-altered, are more common than midbrain germinomas, and primary central nervous system lymphoma is also occasionally found. Indeed, 5 midbrain germinoma cases were preoperatively suspected to be tectal glioma, diffuse intrinsic glioma, and malignant lymphoma.^2,5,6,8^ Another 3 cases were initially treated with steroids, as nonneoplastic lesions such as sarcoidosis and multiple sclerosis were clinically suspected.^3,7,9^ These 3 lesions were not biopsied until 3–4 years after initial detection. Because of the pivotal anatomical structure, even biopsy for midbrain intrinsic tumors is not feasible. Thus, clinical and radiological differentiation and a less invasive diagnosis are crucial for an optimal therapeutic approach.

**Table 1.** Midbrain Germinoma Cases

Age/Sex	Symptom (Duration)	MRI Findings				Surgery	Chemotherapy	Radiotherapy	Outcome	Reference
		Midbrain Lesion	Thalamic Lesion	Enhancement	Cystic Component					
15/M	Diplopia, headache, personality change (ND)	Tegmentum	Bilateral	Enhancement	+	Stereotactic	CDDP + ETP	Local 45 Gy	Alive, 1 year	2
18/M	Headache, vomiting, diplopia (10 months)	Tegmentum Tectum	Bilateral	Homogenous	+	Stereotactic	−	WV 24 Gy + local 16 Gy	Alive, 4 years	7
26/M	Diplopia, ataxia (4 years)	Tegmentum	Bilateral	Homogenous	−	Stereotactic	−	60 Gy	ND	9
27/M	Diplopia (5 years)	Tegmentum	−	Homogenous	−	Stereotactic	−	WB 30 Gy + local 20 Gy	Alive, 1 month	5
29/M	Diplopia (1 year)	Tegmentum	−	Homogenous	−	Stereotactic	CDDP+ETP	Local 40 Gy	Alive, 7 months	3
29/M	Diplopia (8 months)	Tegmentum Tectum	Unilateral	Homogenous	+	Open	CBDCA + ETP	WV 24 Gy	Alive, 5 years	4
39/M	Diplopia, memory disturbance (6 months)	Tegmentum Tectum	Bilateral	Ring	−	Open	CBDCA + ETP	WV 23.4 Gy	Alive, 5 years	6
22/M	Diplopia, headache (ND)	Tegmentum Tectum	Unilateral	Homogenous	+	Endoscopic	CBDCA + ETP	36 Gy	Alive, 6 months	8
25/M	Diplopia (6 months)	Tegmentum Tectum	Bilateral	Ring	+	Endoscopic	CBDCA + ETP	WV 23.4 Gy + local 12.6 Gy	Alive, 1 year	Present case

CBDCA, carboplatin; CDDP, cisplatin; ETP, etoposide; ND, not described; WB, whole brain; WV, whole ventricle.

Clinically, all midbrain germ cell tumor cases were men and 75% (6/9) were Asian.^3–6,8^ The median age at diagnosis was 26 (ranging from 15 to 39) years, which is relatively older than is for germinoma at the pineal and neurohypophysis location.^1^ All cases presented with diplopia, which was more frequent than pineal tumors (approximately half of cases).^11^ Similar to midbrain germinoma, cranial nerve deficits occur in most diffuse intrinsic brainstem glioma, and in about half of tectal glioma cases.^12^ On the other hand, diabetic insipidus was not found to be associated with any midbrain germ cell tumor. Notably, all cases demonstrated relatively long-term symptoms, ranging from 6 months to 5 years. This clearly differs from high-grade tumors. Although it is not feasible to distinguish midbrain germinoma from other brain tumors, these unique clinical features may support differentiating diagnosis.

Through a literature review (Table 1), we found that all reported midbrain germinomas were located mainly at the tegmentum of the midbrain. Seven of the 9 cases showed lesions in the medial thalamus, along with the tegmentum of the midbrain lesions.^2,4,6–9^ Moreover, 5 cases demonstrated butterfly-like radiographic features, which were shown as iso-intensity lesions on T2/FLAIR images with homogenous contrast enhancement in the midbrain. High-intensity lesions on FLAIR were asymmetrically extended to the bilateral thalamus.^2,6,7,9^ These findings are relatively distinctive for midbrain germinoma and different from those of pilocytic astrocytoma in the tectal region and diffuse midline glioma, H3 K27-altered. Pilocytic astrocytoma in the tectal region typically shows high intensity on T2-weighted images and less contrast enhancement. Diffuse midline glioma, H3 K27-altered originates in the pons, and thalamus and also involves the midbrain. However, these tumors showed a diffusely enlarged thalamus or pons with hyperintensity on T2-weighted images.^13^ Additionally, all reported midbrain germinomas demonstrated obvious contrast enhancement; 7 cases showed homogenous enhancement, while the other 2 showed ringed enhancement. These findings are typical of germinomas in common locations. Also, 5 out of 9 cases had cystic components, which is a common finding in basal ganglia-originated germinoma. Taken together, radiographic features, such as T2/FLAIR iso-intensity lesion with homogenous contrast enhancement, tegmentum lesion with asymmetrically extending bilateral thalamus, and cyst formation, are likely common characteristics in midbrain germinomas.

Midbrain germinomas were biopsied and diagnosed using the following approaches (Table 1): stereotactic biopsy in 5 cases,^2,3,5,7,9^ open biopsy via occipital transtentorial approach in 2 cases,^4,6^ and transventricular endoscopic biopsy in 2 cases.^8^ Stereotactic biopsies can easily reach deep lesions, but small sample collection is a critical diagnostic problem.^2,5,7^ In comparison, multiple specimens can be collected by open or endoscopic biopsy. As in our case, multiple biopsies may allow sufficient sampling for pathological diagnosis and genomic analysis which may be required for future diagnostic criteria. Open biopsy using the occipital transtentorial approach is a relatively complicated technique that can be directly performed for lesions located on the dorsal surface of the midbrain. In contrast, the endoscopic approach may be feasible in cases where the midbrain germinoma spreads to the medial side of the thalamus. Since 78% of the cases involve medial thalamic lesions,^2,4,6–9^ the endoscopic approach might be reasonable for most midbrain germinomas. In addition, the endoscopic approach allows a third ventriculostomy in cases of obstructive hydrocephalus. Therefore, the endoscopic approach may be advantageous for safety sampling in the midbrain germinomas.

We performed chemotherapy using carboplatin and etoposide with whole-ventricular irradiation and a local boost, and achieved a complete response. Intracranial germinomas have a favorable prognosis in patients treated with a combination of chemotherapy and radiotherapy. However, because whole-ventricle irradiation covers a 0.5- to 1.0-cm margin around the ventricles,^1^ the entire midbrain lesion may not be covered in some cases. Because most germinoma recurrences are outside the irradiation field, whole-brain irradiation may be an alternative strategy for midbrain germ cell tumors. Previous reports have shown a favorable prognosis using either whole-ventricle or local or whole-brain irradiation; however, long-term prognosis beyond 5 years has not been described (Table 1). As germinomas demonstrate a 5-year progression-free rate of approximately 90%,^1^ long-term follow-ups are required to determine the optimal chemotherapeutic regimen and irradiation strategy for germ cell tumors arising in the midbrain.

This is the first case of midbrain germinoma with comprehensive genetic and DNA methylation analysis. Germinomas are characterized by global DNA hypomethylation and frequently harbor mutations in the MAPK pathway and/or PI3K pathway.^14,15^ This case indicates that genetic and epigenetic features are similar to germinomas arising from common locations. Migrating primordial germ cells have been shown to acquire these alterations, and precursor cells migrating to the diencephalon typically differentiate into the pineal and neurohypophyseal germinomas.^14^ In contrast, our findings support the hypothesis that precursor cells with common genomic and epigenetic alterations migrate to the mesencephalon, resulting in the development of midbrain germinomas. Based on the radiographic findings of pineal calcification outside the tumor and an intact pineal gland based on intraoperative findings, we speculated that the pineal region may be normal, and we did not perform a biopsy from the pineal body. Therefore, we could not conclude that the tumor was completely distinct from the pineal region. Nonetheless, these findings suggest that the germinoma in this case may have originated outside the pineal body. Further studies are required to elucidate the tumor origin, which may provide a novel therapeutic strategy for midbrain germinomas.

In summary, midbrain germinoma is a rare disease that should be considered if clinical and radiological findings are typical. Once midbrain germinoma is suspected, an endoscopic biopsy may be ideal to assess thalamic and midbrain lesions. Further molecular and radiographic studies may support optimal therapeutic strategy for midbrain germinoma.

## Supplementary Material

vdad043_suppl_Supplementary_Tables

vdad043_suppl_Supplementary_Figures

vdad043_suppl_Supplementary_Video_S1
