# Supplementary material for: A case of midbrain germinoma: A literature review for radiographic and clinical features
Source: Neurooncol Adv. 2023 Apr 19;5(1):vdad043. doi: 10.1093/noajnl/vdad043 (PMC10195201; doi:10.1093/noajnl/vdad043)
Supplement: vdad043_suppl_Supplementary_Figures [file vdad043_suppl_supplementary_figures.pdf]

Supplementary Figure 1

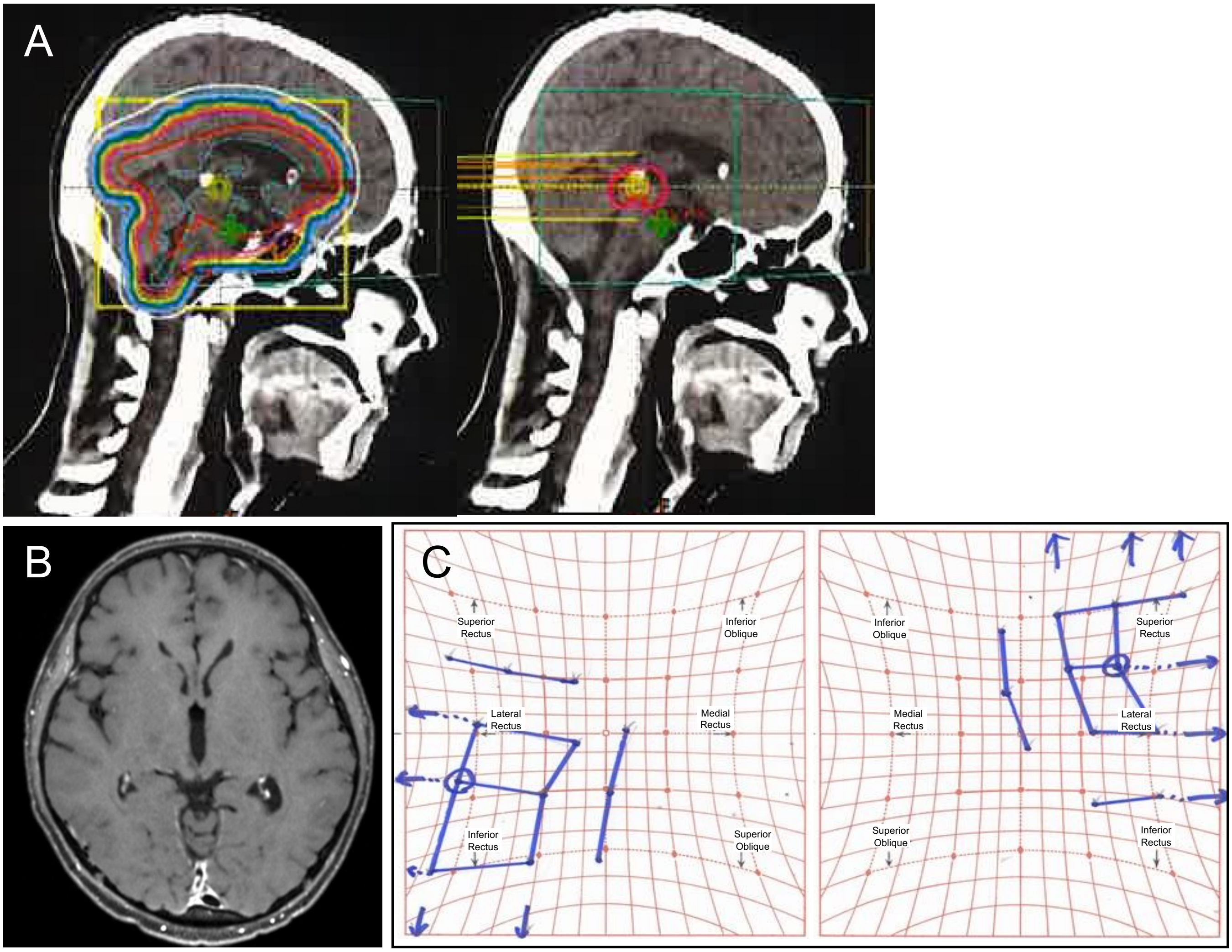

Supplementary Figure 1. **A**: Irradiation fields of the whole ventricle (left) and focal (right). Redline showing planning target volume. **B**: Gadolinium (Gd)-enhanced T1-weighted imaging after adjuvant treatment. **C**: Hess screen test after adjuvant treatment.

Supplementary Figure 2

A

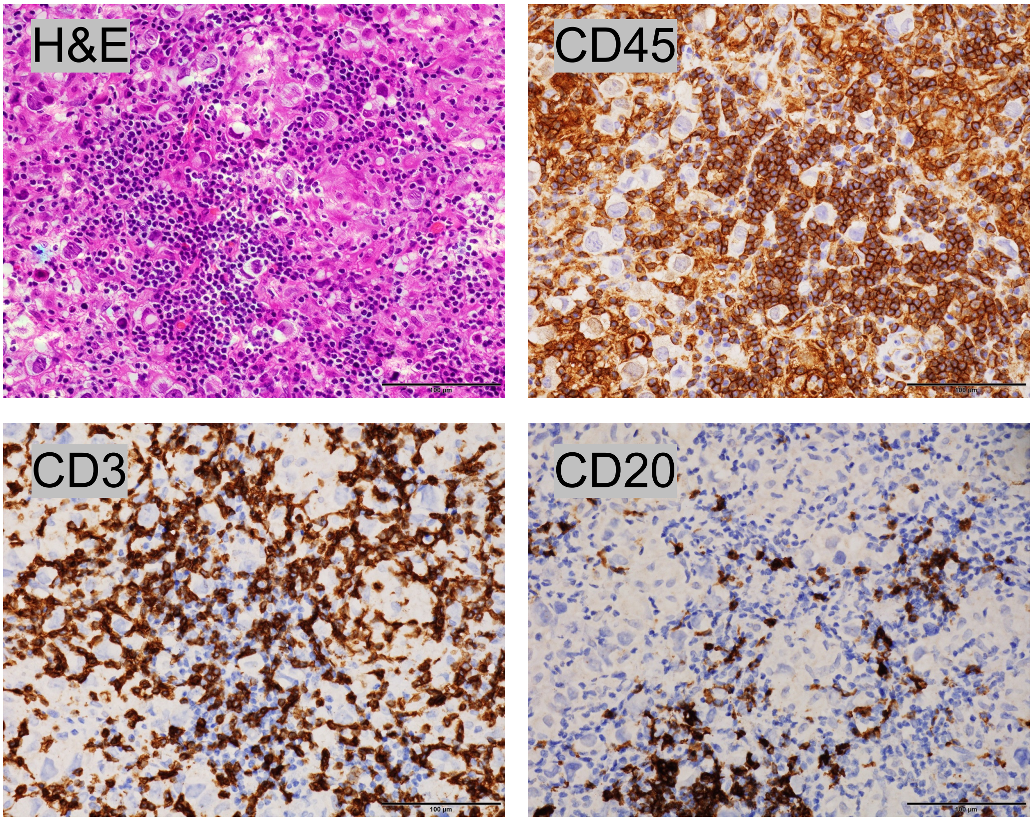

B

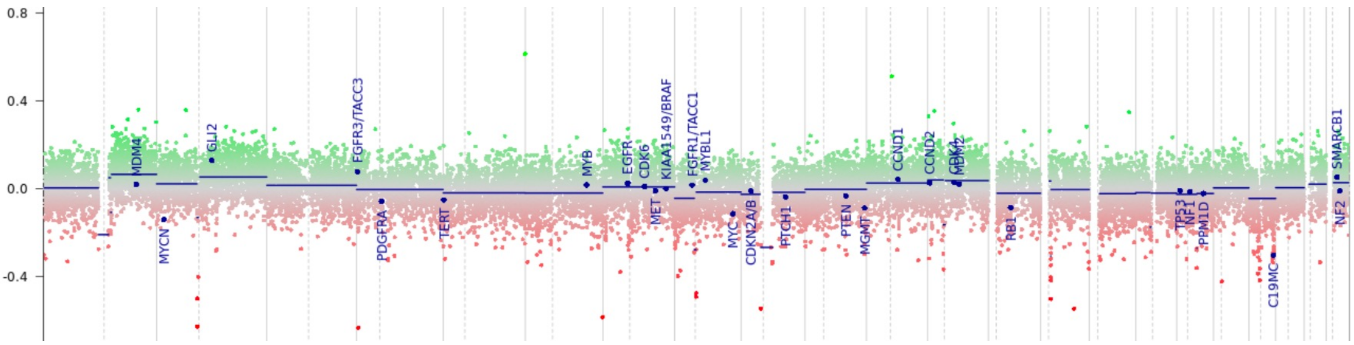

Supplementary Figure 2.

A: Hematoxylin and eosin staining and Immunohistochemistry for indicated proteins in YMG206 tumor. Bars, 100µm. **B:** Copy number profiling of YMG206 tumor, as indicated by DNA methylation data.
